# Web-Based Intervention Using Behavioral Activation and Physical Activity for Adults With Depression (The eMotion Study): Pilot Randomized Controlled Trial

**DOI:** 10.2196/10112

**Published:** 2018-07-16

**Authors:** Jeffrey D Lambert, Colin J Greaves, Paul Farrand, Lisa Price, Anne M Haase, Adrian H Taylor

**Affiliations:** ^1^ Institute for Health Research Primary Care University of Exeter Medical School Exeter United Kingdom; ^2^ School for Sport, Exercise and Rehabilitation University of Birmingham Birmingham United Kingdom; ^3^ Clinical Education, Development and Research Psychology University of Exeter Exeter United Kingdom; ^4^ Sport and Health Sciences University of Exeter Exeter United Kingdom; ^5^ Centre for Exercise, Nutrition and Health Sciences University of Bristol Bristol United Kingdom; ^6^ Faculty of Medicine & Dentistry University of Plymouth Plymouth United Kingdom

**Keywords:** psychological therapy, mood, anxiety, exercise, eHealth, feasibility, acceptability

## Abstract

**Background:**

Physical activity is a potentially effective treatment for depression and depressive relapse. However, promoting physical activity in people with depression is challenging. Interventions informed by theory and evidence are therefore needed to support people with depression to become more physically active. eMotion is a Web-based intervention combining behavioral activation and physical activity promotion for people in the community with symptoms of depression.

**Objective:**

The objectives were to assess the feasibility and acceptability of delivering eMotion to people in the community with symptoms of depression and to explore outcomes.

**Methods:**

Participants with elevated depressive symptoms were recruited from the community through various methods (eg, social media) and randomized to eMotion or a waiting list control group for 8 weeks. eMotion is an administratively supported weekly modular program that helps people use key behavior change techniques (eg, graded tasks, action planning, and self-monitoring) to re-engage in routine, pleasurable, and necessary activities, with a focus on physical activities. Feasibility data were collected that included the following: recruitment and trial retention rates; fidelity of intervention delivery, receipt, and enactment; and acceptability of the intervention and data collection procedures. Data were collected for the primary (depression) and secondary outcomes (eg, anxiety, physical activity, fidelity, and client satisfaction) at baseline and 2 months postrandomization using self-reported Web-based questionnaires and accelerometers. Delivery fidelity (logins, modules accessed, time spent) was tracked using Web usage statistics. Exploratory analyses were conducted on the primary and secondary outcomes.

**Results:**

Of the 183 people who contacted the research team, 62 were recruited and randomized. The mean baseline score was 14.6 (SD 3.2) on the 8-item Patient Health Questionnaire depression scale (PHQ-8). Of those randomized, 52 participants provided accelerometer-recorded physical activity data at baseline that showed a median of 35.8 (interquartile range [IQR] 0.0-98.6) minutes of moderate-to-vigorous physical activity (MVPA) recorded in at least 10-minute bouts per week, with only 13% (7/52) people achieving guideline levels (150 minutes of MVPA per week). In total, 81% (50/62) of participants provided follow-up data for the primary outcome (PHQ-8), but only 39% (24/62) provided follow-up accelerometer data. Within the intervention group, the median number of logins, modules accessed, and total minutes spent on eMotion was 3 (IQR 2.0-8.0), 3 (IQR 2.0-5.0), and 41.3 (IQR 18.9-90.4), respectively. Acceptability was mixed. Exploratory data analysis showed that PHQ-8 levels were lower for the intervention group than for the control group at 2 months postrandomization (adjusted mean difference −3.6, 95% CI −6.1 to −1.1).

**Conclusions:**

It was feasible to deliver eMotion in UK communities to inactive populations. eMotion has the potential to be effective and is ready for testing in a full-scale trial. Further work is needed to improve engagement with both the intervention and data collection procedures.

**Trial Registration:**

ClinicalTrials.gov NCT03084055; https://clinicaltrials.gov/ct2/show/NCT03084055 (Archived by WebCite at http://www.webcitation.org/6zoyM8UXa)

## Introduction

Depression has a significant detrimental impact on individuals and their families as well as being associated with increased utilization of health services and reduced productivity at work. In the UK, the cost to the economy due to sickness absence, staff turnover, benefits, fall in tax revenue, and costs to the National Health Service (NHS) is estimated to be between £74 billion and £99 billion per year [1]. Depression is also associated with a range of major physical illnesses (which are also associated with physical inactivity), including diabetes [2,3], coronary heart disease [4], and obesity [5].

Physical activity (PA) has been shown to be effective in treating [6-8] and preventing [9] depression and is often cited by patients as their preferred treatment option [10,11]. A recent systematic review of randomized controlled trials (RCTs) found PA to be more effective than control conditions for reducing depression (standardized mean difference [SMD] −0.62) and just as effective as pharmacotherapy (SMD −0.11) and psychological therapies (SMD −0.03) [6]. PA also has the potential to reduce depressive relapses [12], improve anxiety symptoms [13], and help prevent many physical health problems associated with depression such as cardiovascular disease, cancer, and diabetes [14]. Despite these benefits, PA is perceived as difficult to prescribe compared with medication [15]. Further studies are therefore needed to understand how and whether PA can be cost-effectively used to improve outcomes for people with depression, especially in those who are relatively inactive [16].

Behavioral activation (BA) is an evidenced-based psychological therapy. BA focuses on reducing an individual’s exposure to sources of negative reinforcement (ie, short-term relief from avoiding burdensome activities) while increasing experiences of positive reinforcement (ie, social and personal activities that bring pleasure and achievement), leading to reduced avoidant type-behaviors in the future [17-19]. A meta-analysis of 26 RCTs (n=1524) conducted on adults with depression found BA to be superior to usual care, wait list, or placebo control conditions (SMD −0.74) and medication (SMD −0.42) for reducing depression [20]. The treatment rationale of BA shares behavior change techniques (BCTs) [21] with interventions promoting PA (eg, goal setting) [22]. By subtly shifting the behavioral emphasis, the treatment rationale of BA could therefore provide a useful delivery mechanism for promoting PA in people with depression, capitalizing on the dual benefits of PA and BA. Furthermore, sustained PA may reduce the relapse rate associated with BA alone due to its inherent mood enhancing and long-term benefits [12].

Recent studies have examined the feasibility of delivering a combined BA and PA intervention (BAcPAc) within the context of existing mental health services [22,23]. However, BAcPAc was difficult to implement in overstretched services, it was hard to recruit patients, and problems of fidelity were observed, with providers not delivering the treatment as intended [23]. Web-based interventions could provide a useful way of overcoming these limitations by delivering such interventions outside of existing services, recruiting directly from the community, and standardizing fidelity [24,25]. Furthermore, Web-based interventions could have additional benefits by being scalable, cheap, and accessible [26]. Importantly, Web-based interventions can also be used to provide support to people experiencing depression in UK communities who do not seek help from health services due to social stigma and identity conflict [27]. However, despite the potential benefits associated with Web-based interventions, there remains a paucity of studies that have attempted to deliver Web-based interventions promoting PA for depression [28-31]. Furthermore, none of these studies have explored the feasibly of delivering a Web-based intervention combining BA and PA for people with depression.

The purpose of the present study was to examine the feasibility of delivering a Web-based intervention (eMotion), combining PA with BA, for people with depression and to explore its effects on depression and PA. The key aims of the study were to: explore participant recruitment and attrition rates throughout the study; explore the feasibility and acceptability of data collection and study procedures; examine baseline characteristics of the recruited sample, including levels of PA; explore the fidelity of delivery, receipt, and enactment (use of techniques) of eMotion and its acceptability to participants; and estimate and explore the variance in key outcomes (depression scores and PA).

## Methods

### Trial Design

#### Description of Trial Design

The eMotion trial was a 2-arm, individually randomized, parallel-group pilot RCT with a nested process evaluation (ClinicalTrials.gov Identifier: NCT03084055). The study was reported in accordance with Consolidated Standards of Reporting Trials (CONSORT-EHEALTH) recommendations for reporting of RCTs of eHealth interventions [32] and the Template for Intervention Description and Replication (TIDieR) recommendations on reporting of behavior change interventions [33].

#### Important Changes to Methods After Trial Commencement

The eMotion trial provided “minimal contact” administrative support [34] at week 2 of the intervention to provide the participant with a rationale for the use of self-help materials and check-ins related to progress, but with no focus on any clinical or behavior change issues. This support was initially intended to be provided by an independent “supporter.” However, due to resource issues, this support was provided by the lead author.

### Participants

#### Eligibility Criteria for Participants

Participants were eligible for the study if they were more than 18 years old, were living in UK, had at least moderate depressive symptoms [defined as scoring at least 10 on the 8-item Patient Health Questionnaire depression scale (PHQ-8)], had access to the internet and were computer literate, reported being able to walk continuously and unaided for a minimum of 5 min, and provided informed consent to participate. Eligible adults were recruited from the community via advertisements in weekly newspapers, social media (eg, Facebook support groups, Twitter), and through banners on websites relating to mental health problems. All adverts contained the primary investigator’s contact details. Potential participants did not need to be referred by a general practitioner or mental health care practitioner. After contacting the lead author by phone or email, potential participants were sent the participant information sheet (PIS), consent form, and a link to the Web-based screening questionnaire via email. At this point, they were informed that they could withdraw from the study at any time without consequence or being obliged to provide a reason. Once participants read the PIS, they were asked to complete the Web-based consent form (indicating consent using a checkbox) followed by a screening questionnaire used by the lead author to assess participant eligibility. After screening, the lead author contacted the participant via phone to clarify the study procedures and provide instructions for wearing the wrist-worn accelerometers sent by post. The participant was instructed to wear the accelerometer for 7 days and return it in a pre-stamped addressed envelope. However, participant refusal to wear an accelerometer did not preclude randomization. Further baseline measures were then administered via a separate Web-based questionnaire. Participants were not paid for their involvement in the study.

### The eMotion Intervention

The eMotion intervention is a Web-based course that provides people with access to an evidence-based treatment based on BA with added PA promotion. People with depression commonly reduce activities that they perceive as burdensome, making less effort to do things they may have previously enjoyed. By avoiding such activities, people with depression experience temporary relief that then negatively reinforces the likelihood of avoiding further activities. However, avoiding such activities has a long-term cost because it reduces the opportunities for positive reinforcement that occurs when people engage in social and personal activities that bring them pleasure and achievement [17-19]. PA is often avoided by people with depression, but it has the potential to provide additional anti-depressive benefits as well as added health benefits.

Through a series of steps delivered in a week-by-week modular fashion (Table 1), eMotion teaches people how to re-establish daily routines, increasing activities that provide positive reinforcement while reducing negative reinforcement. eMotion comprises 13 modules (1 introduction module, 8 weekly modules, 1 generic problem-solving module, and 3 unlockable modules) consisting of visual content with an audio voiceover triggered when each slide opens. Printable, interactive worksheets, and emails were also included, with links to the slides to allow downloading to a personal computer or another device (eg, tablet or smartphone). Automated reminder emails were also sent once a week by the eMotion program following registration. Where possible, brief administrative/motivational support via a 10-minute phone call was provided at week 2.

Key content related to the rationale of BA was front loaded in the introduction, week 1, and week 2 and was considered the “minimum dose.” The remaining weekly modules (weeks 3-8) were shorter and designed to support people to review and update their plans. The intervention supports “effective engagement” and self-regulation by encouraging people to review their plans weekly, irrespective of whether or not they continue to login (eg, using their own diaries).

### eMotion Development

The content for eMotion was developed by the study authors (JDL, CJG, PF, AMH, and AHT). The Living Life to the Full Web-based platform was used to host the intervention. eMotion was adapted from the BAcPAc intervention [22,23] using the Centre for eHealth Research and Disease Management (CeHReS) roadmap [35]. The CeHReS roadmap is intended to help the planning, coordination, and execution of the participatory development process of eHealth. In eMotion, this involved using patient and public involvement, usability testing, and a structured literature search. A full description of the eMotion intervention and its developmental process was previously provided [36].

### Control (Waiting List)

Participants in the control group did not receive the eMotion intervention but were able to access usual care as normal. After data collection at the 2-month time point, participants were given access to and instructions for using eMotion. No eMotion facilitator support was provided, although participants could contact the lead researcher for (nonclinical) support if they experienced any difficulties using the intervention.

**Table 1:** eMotion structure.

Steps	Content	Module in eMotion
Step 1: Provide a rationale	People are provided with a full and comprehensive rationale for behavioral activation and PA^a^, including reference to the interaction of physiological, behavioral, and cognitive emotional symptoms, the role of avoidance in maintaining low mood and the idea of routine, pleasurable, necessary, and activities (including PA).	Introduction and Week 1
Step 2: Identify activities	People are helped to identify routine, pleasurable, and necessary activities (including PA)—things that they would like to do but have usually stopped doing since they became depressed.	Weeks 2-8
Step 3: Make a hierarchy of activities	People are helped to organize the activities into a hierarchy of difficulty—most difficult, medium difficult, and easiest. People should include some of each type of routine, pleasurable, and necessary activity (including PA).	Weeks 2-8
Step 4: Plan some activities	eMotion helps people to schedule some avoided activities into their week, to specify a mixture of routine, pleasurable, and necessary activities (including PA). These should be initially identified from the “easiest” category of their hierarchy from step 3). Activities should be detailed precisely: what, where, when, and with whom.	Weeks 2-8
Step 5: Implement activities	People are encouraged to undertake the planned activities. The principle of grading activities and using a mixture of routine, pleasurable, and necessary activities (including PA) should be followed. People should record if they accomplished the planned activity.	Weeks 2-8
Step 6: Review progress	People are encouraged to reflect on their progress, congratulating themselves for success and overcoming any problem-solving difficulties experienced during implementation. People may make sporadic progress and activities may not go as planned.	Weeks 3-8

^a^PA: Physical activity.

### Feasibility Outcomes

#### Recruitment and Attrition

Participant recruitment rates were derived by calculating the absolute number of people randomized in the trial relative to those who expressed an initial interest in the study. Participant attrition was defined as the percentage of randomized participants who began the intervention but failed to provide primary outcome data (PHQ-8) at the 2-month data collection point.

#### Feasibility of Data Collection

Feasibility of data collection was explored by assessing the percentage of Web-based screening, baseline, and 2-month postrandomization questionnaires that were completed, as intended. For accelerometers, the percentage of devices that were returned at baseline and at 2 months follow-up as well as the amount of valid wear time were assessed. Reasons for any missing data were tabulated, where available.

### Primary Outcome for the Planned Future Trial

#### Depression

The PHQ-8 was delivered at screening and at 2 months postrandomization using a Web-based self-completed version of the questionnaire. The PHQ-8 is a freely available 8-item self-report measure based on the symptoms of depression described in the Diagnostic and Statistical Manual for Mental Disorders (DSM-IV). It measures the frequency of depressive symptoms over the preceding 2-week period. A score of at least 10 on the PHQ-8 has a positive likelihood ratio of 28 for detecting major depression (ie, a patient with any depressive disorder is 28 times more likely to have a PHQ-8 score of 10-24 than someone without a depressive disorder) [37]. Each item is rated on a scale of 0-3, producing a range of scores from 0-24 (0-4=no depression, 5-9=mild depression, 10-14=moderate depression, 15-19=moderately severe depression, and 20-24=severe depression). The PHQ-8 has good validity, reliability, sensitivity, and specificity [37] and has been used in previous Web-based intervention studies of low mood and depression [38].

### Secondary Outcomes for the Planned Future Trial

#### Objective Physical Activity

GENEActiv accelerometers (Activeinsights Ltd., Kimboloton, Cambs, UK) were used to record PA at baseline and at 2-months postrandomization. The GENEActiv is a small wrist-worn device that measures and records acceleration. It was set to record at 100Hz. Data were downloaded using the GENEActiv PC software (version 2.9) and processed in R (https://cran.r-project.org/) using package GGIR (version 1.2-8) [39,40]. Raw data were used to create a vector magnitude √(x^2+ y^2+z^2)-1g negative numbers were rounded to 0 to create the Euclidean Norm minus one (measured in mg), as previously reported [41]. Data were averaged over 5-s epochs. Nonwear was assessed over a 60-minute window, using moving 15-minute increments [42] if the standard deviation of 2 of the 3 axes were less than 13 mg and the value range was less than 50 mg. Participants were mailed the device before randomization and instructed to wear it continuously on their nondominant hand for 7 days from the following morning, without changing their routine PA. To be considered valid for analysis, data were needed for at least 4 days with a minimum of 10 h per day, including at least 1 day on the weekend. Published thresholds were used to determine average daily minutes of activity in light (LPA), moderate (MPA), vigorous (VPA), and moderate and vigorous (MVPA) intensities [43]. Minutes of activity accumulated in 10-minute bouts were established using an 80% rule, where activity must be sustained above the appropriate threshold for at least 80% of the time [42].

#### Self-Reported Physical Activity

Minutes per week of MVPA were estimated using Web-based self-completion of the International PA Questionnaire-Short Form (IPAQ-SF) at baseline and at 2-months postrandomization. The IPAQ-SF is a validated measure of PA [44] and has been used in previous behavioral trials promoting PA for depression [29,45] as well as being the most frequently used measure in Web-based studies for PA [46].

#### Anxiety

The General Anxiety Disorder scale (GAD-7) is a 7-item, 4-point scale (0-3) and was used to assess anxiety using Web-based self-completion at baseline and at 2-months postrandomization. The GAD-7 measures the severity of anxiety symptoms over the past 2 weeks based on the DSM-IV criteria. The GAD-7 has good reliability as well as criterion, construct, factorial, and procedural validity. At the cutoff point of 10, the GAD-7 has a sensitivity of 89% and specificity of 82% [37].

#### Demographic Data

Data on age, gender, level of education (GCSE, A-levels, degree, postgraduate, or doctoral), employment status (full-time, part-time, homemaker, student, retired, or unemployed), current receipt of psychotherapy (yes or no), current receipt of antidepressants (yes or no), method of recruitment (social media, newspaper, word of mouth, or other), and ethnicity were collected at baseline using a Web-based questionnaire.

#### Fidelity

As recommended in previous studies [25,47], intervention fidelity was conceptualized and measured in the domains of design fidelity, training fidelity, quality/completeness of delivery, participant receipt, and enactment. The process of establishing good design fidelity for the eMotion intervention was previously reported [36]. However, given that the eMotion intervention had very limited external human support, training fidelity was not applicable in this study. Delivery fidelity was assessed using website usage statistics from the Web-based intervention database. This database provided individual level data about whether the participant registered for eMotion, modules accessed, and the total time spent on each module. Participant receipt and enactment of the intended intervention processes were measured using Web-based questionnaires. For fidelity of receipt, 2 approaches were used. The first approach assessed participants’ understanding of how emotions, behaviors, thoughts, and physical feelings affect each other to maintain depression over time. A single item was used based on questions used in a previous study [48]. The item employed a 5-point Likert response scale (Strongly Agree to Strongly Disagree) assessing participant agreement with the following statement: “I understand how emotions, behaviors, thoughts, and physical feelings affect each other to maintain depression over time.” The second approach assessed participants’ perceived ability to use the intended BCTs. This was assessed by asking participants to rate their confidence in using specific BCTs (ie, identification of suitable activities, grading activities for ease of use, and planning and dealing with setbacks) over the last 2 months on a scale from 1 (not at all confident) to 10 (very confident). This measure was adapted from measures of confidence used in the ProActive trial [49]. Finally, to assess enactment, we asked participants if they had used specific BCTs related to BA in the last 2 months using a binary scale (yes/no). This measure was adapted from similar measures of BCT usage showing that enactment was significantly associated with weight loss, providing initial evidence of the validity of this type of measure [50].

#### Acceptability

The Client Satisfaction Questionnaire-Short Form (CSQ-SF) is a 4-item measure and was used to assess participant satisfaction regarding their use of eMotion 2 months postrandomization (given to intervention participants only). This measure was administered using a Web-based questionnaire and has been used to assess treatment satisfaction in other studies of Web-based interventions for depression [51].

### Sample Size

Due to the pilot nature of the study, no formal sample size calculations were conducted. However, to ensure a suitably reliable estimate of the standard deviations to power a future trial with 90% power, at least 15 people per arm were recommended if the expected effect size was to be between 0.3 and 0.7 [52]. A previous meta-analysis of computer-based psychological treatments for depression reported a moderate effect size (0.56) and a drop-out rate of 57% [38]. As such, a target sample size of 62 was adopted (accounting for a possible attrition rate of 50%) to ensure at least 15 people per arm at follow-up.

### Randomization

Once participants completed the baseline assessment, they were randomly allocated to either the intervention or control group using simple randomization at the individual level in a 1:1 ratio and a Web-based randomization service (Sealed Envelope Ltd. 2016). Personal details were anonymized through the use of participant numbers that were entered into the website by the lead author in a consecutive manner (in the order of completed baseline assessment), and the randomization service allocated them to either group A (eMotion) or group B (waiting list) without any stratification.

### Blinding

Due to limited resources for the study, the lead author was not blinded to which condition each participant was allocated following randomization. Due to the nature of the intervention, it was also impossible to blind participants to group allocation. However, because outcome measures were taken using Web-based self-report surveys, there was a reduced chance of the lead author influencing the participant’s responses or for the lead author to misinterpret responses or introduce subjective bias into recorded observations [53].

### Statistical Analysis

Quantitative methods were used to explore the following: recruitment and attrition rates of trial participants; feasibility of data collection and study procedures; baseline data (including levels of PA and baseline differences between groups and between dropouts); and fidelity of delivery. Descriptive statistics were produced for all outcomes by trial arm at baseline and 2-month follow-up. All quantitative analyses were conducted using Stata SE statistical software release 14 (StataCorp. 2015; College Station, TX). No formal hypothesis testing relating to primary outcomes was planned because this was a pilot study. However, descriptive statistics were used to assess recruitment and retention rates and baseline PA levels. Baseline demographic and clinical characteristics were descriptively presented as proportions or as means with standard deviations. Two types of exploratory analyses of the primary outcome (PHQ-8) were conducted: 1) linear regression models to report changes in depression with 95% confidence intervals around the between-group mean difference and 2) logistic regression models that dichotomized the primary outcome to reflect clinically meaningful change (a reduction to below 10 on the PHQ-8 indicated that the person may no longer qualify for major depression) [37]. The analyses were conducted on participants with complete data only, which included those who began treatment and provided follow-up data regardless of treatment compliance. Missing data were not imputed. Similar analyses were conducted for anxiety, objective, and self-reported PA. We conducted sensitivity analyses using linear regression models to examine the effects of receiving psychological therapies as well as any substantial differences in baseline characteristics on the findings. We also analyzed the mean reduction in depressive symptoms for those who received the minimum dose of intervention and provided data at 2 months postrandomization.

### Ethics

This study was approved by the University of Exeter Sports and Health Sciences Research Ethics Committee (AM160316-21 151021/B/03). One possible ethical issue in this study was suicide risk in people experiencing depression. Because this was a research study on a nonclinical sample, all participants were advised on the PIS that the study was not a clinical or NHS treatment and that the University and researchers could not take clinical responsibility for the treatment of any conditions they might have including depression. They were also signposted to other appropriate resources in case they wished to seek formal treatment. If, at any point in the study (eg, while on the phone to a researcher during screening or after inclusion), participants indicated suicidal intent, the University of Exeter Mood Disorders Suicide Risk Protocol was invoked.

## Results

### Participant flow

**Figure 1:**
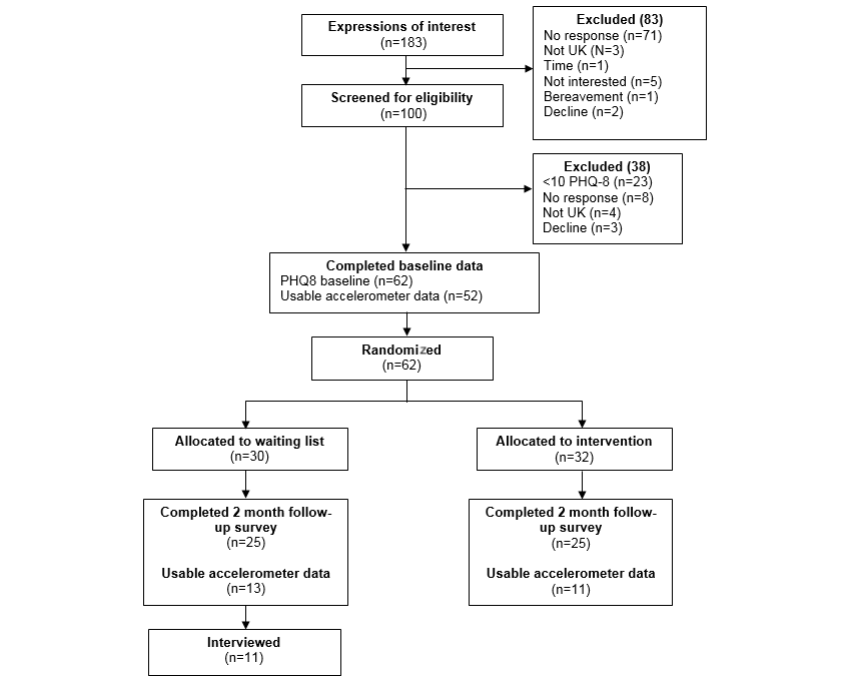
CONSORT flow chart.

A total of 183 people responded to the adverts, with 100 completing screening for eligibility (Figure 1). Of the 183 individuals who initially inquired about the study, 100 were still interested and screened for eligibility and 62 (34% of those who initially enquired [95% CI 27-41] and 62% (62/100) of those who were screened [95% CI 52-71]) were eligible for inclusion and randomized in the trial between May 2016 and February 2017 (32 in the eMotion group, 30 in the control group). Overall attrition in relation with the planned main trial primary outcome (PHQ-8) at 2 months postrandomization was 19% (12/62; 95% CI 11-31). Of those randomized, 94% (58/62) of participants provided complete secondary outcome baseline measurements (eg, GAD7, IPAQ-SF) and 84% (52/62) provided usable accelerometer data at baseline. At 2-month follow-up, 81% (50/62) of those randomized [95% CI 71-91] provided PHQ-8 (and other survey data) and 39% (24/62) of those randomized [95% CI 27-52] provided valid accelerometer data. Only 76% (47/62) and 53% (33/62) participants provided valid IPAQ-SF data at baseline and 2 months postrandomization, respectively. This lack of usable IPAQ-SF data was due to people providing invalid responses.

### Feasibility of Accelerometer Data Collection

At baseline, 6 accelerometers were not sent out due to participants not responding to the request confirming their willingness to wear it or not being willing /able to wear it. Three accelerometers had data processing problems (technical failure). At follow-up, missing accelerometer data were primarily due to participants not responding to the request to wear the device again (n=13) or participants being lost to follow-up (not responding in any way; n=9).

### Baseline Demographic and Clinical Characteristics

At baseline (Table 2 and Table 3), the mean age was 38 years with women accounting for 84% (52/62) of all participants, and 97% (60/62) of participants were white British. Nearly half the sample was recruited through social media (Facebook or Twitter) with the second most popular method being “word of mouth” (ie, hearing about the study from friends or family). Participants had a range of educational levels, and most (55/62, 89%) of them were employed either part-time or full-time. The mean score on the PHQ-8 was 14.6 (SD 3.2), and the mean score on the GAD-7 was 11.8 (SD 4.5). All PA data were positively skewed; hence, medians and interquartile ranges were reported. The median daily total minutes of accelerometer-measured PA was 174.3 (IQR 136.8-212.5) for light PA (LPA), 53.5 (IQR 39.8-80.7) for moderate PA (MPA), 2.9 (IQR 1.0-6.2) for vigorous PA (VPA), and 55.2 (IQR 40.9-90.7) for moderate and vigorous PA (MVPA). The median weekly total minutes of accelerometer-measured MVPA in at least 10-minute bouts was 35.8 (IQR 0.0-98.6). Only 13% (7/52) people achieved at least 150 minutes per week of MVPA in at least 10-minute bouts. The median level of daily self-reported MVPA was 12.9 minutes (IQR 0.0-25.7). Over half (36/62, 58%) of the participants were receiving antidepressants, and 13% (8/62) were receiving some form of psychotherapy, with a higher proportion receiving therapy in the control group (7/30, 23%) than in the intervention group (1/32, 3%). The intervention group had a higher median of total MVPA per day (71 min, IQR 46.7-85.9) than the control group (55 min, IQR 40.1-90.7). Finally, the intervention group was older by 2 years with a mean of 39.3 (12.0) years compared with 36.9 (12.6) years in the control group.

**Table 2:** Participant demographic and clinical characteristics at baseline.

Characteristics	eMotion		Control group		Whole sample	
	N	Mean (SD)	N	Mean (SD)	N	Mean (SD)
Age in years	32	39.3 (12.0)	30	36.9 (12.6)	62	38.1 (12.3)
Depression (PHQ-8^a^)	32	14.4 (3.5)	30	14.8 (2.9)	62	14.6 (3.2)
Anxiety (GAD-7^b^)	31	11.5 (4.7)	27	12.3 (4.2)	58	11.8 (4.5)
Min per week of objective MVPA^c^ in 10-min bouts^d^	27	29.5 (0.0-98.8)	25	42.1 (8.1-93.7)	52	35.8 (0.0-98.6)
IPAQ-SF daily min of MVPA^d^	27	12.9 (0.0-25.7)	20	10.7 (3.6-17.9)	47	12.9 (0.0-25.7)

^a^PHQ-8: Patient Health Questionnaire 8.

^b^GAD-7: General Anxiety Disorder scale.

^c^MVPA: moderate-to-vigorous intensity physical activity.

^d^Data were positively skewed, so medians (interquartile ranges) are reported.

**Table 3:** Participant demographic and clinical characteristics at baseline.

Characteristics	eMotion		Control Group		Whole Sample	
	N	n (%)	N	n (%)	N	n (%)
Female	32	26 (81)	30	26 (87)	62	52 (84)
Receiving therapy	32	1 (3)	30	7 (23)	62	8 (13)
Antidepressants	32	18 (56)	30	18 (60)	62	36 (58)
>150 m per week of MVPA^a^ (10-min bouts)	27	3 (11)	25	4 (16)	52	7 (13)
Currently employed, studying, or training	32	28 (88)	30	27 (90)	62	55 (89)
Educated to A level or beyond	32	25 (78)	30	23 (77)	62	48 (77)

^a^MVPA: moderate-to-vigorous intensity physical activity.

### Intervention fidelity

In total, 88%(28/32) of the intervention participants registered for eMotion and began the introduction module. The median number of logins, modules accessed, and total minutes spent on eMotion was 3 (IQR 2-8), 3 (IQR 2-5), and 41.3 (IQR 18.9-90.4), respectively. Overall, 53% (17/32) of participants completed at least the introduction, week 1, and week 2, and 25% (8/32) of participants completed up to at least week 4. Only one participant used every module. Of the 46 participants who provided receipt and enactment data at both baseline and 2 months postrandomization, those randomized to the eMotion group reported a significant difference, compared with the control group, in levels of understanding about how thoughts, feelings, and behaviors affect mood (adjusted mean difference 0.5, 95% CI −0.0 to −1.0). Significant differences were also found for confidence to identify (adjusted mean difference 1.4, 95% CI 0.0-2.8), select (adjusted mean difference 1.3, 95% CI −0.02 to 2.6), and plan (adjusted mean difference 1.8, 95% CI 0.5-3.1) achievable activities to improve mood as well as confidence to deal with setbacks (adjusted mean difference 1.5, 95% CI 0.2-2.7). Of the participants who answered “no” on the enactment questionnaires at baseline, those who were randomized to the eMotion group were significantly more likely to select (N=25; OR 10, 95% CI 1.6-62.7) and plan (N=33; OR 10.3, 95% CI 2.0-52.6) activities to improve their mood at the 2-month follow-up.

### Acceptability

Of the participants randomized to receive the eMotion intervention who provided follow-up data, 4% (1/25) felt almost all their needs had been met, 32% (8/25) felt most of their needs had been met, 52% (13/25) felt only a few of their needs had been met, and 12% (3/25) felt none of their needs had been met. Twenty-four percent (6/25) said they would definitely use the program again, 32% (8/25) said “Yes I think so,” 40% (10/25) said “No, I don’t think so,” and 4% (1/25) said “Definitely not.” Finally, 16% (4/25) said they were “Very Satisfied,” 40% (10/25) said they were “Mostly Satisfied,” 40% (10/25) said they were “Indifferent or Mildly Satisfied,” and 4% (1/25) said they were “Quite Dissatisfied.”

### Exploratory Analysis of Outcomes

Exploratory analyses carried out on complete data (Table 4) showed that at 2 months postrandomization, the intervention group had a larger reduction in depressive symptoms than the control group (adjusted mean difference −3.6, 95% CI −6.1 to −1.1). In the intervention group, 56% (14/25) of depression scores went below the threshold of 10 on the PHQ-8, compared with 28% (7/25) in the control group (OR 3.3, 95% CI 1.0-10.6). For those who completed the minimum dose of intervention and provided data at 2 months postrandomization (n=15), the mean reduction in depressive symptoms was 5 points (SD 5.4). For those who did not complete the minimum dose of intervention and provided data at 2 months postrandomization (n=9), the mean reduction in depressive symptoms was 4.9 points (SD 4.6). Of the 47 participants who provided anxiety scores at both baseline and 2 months postrandomization, there was a larger reduction in symptoms of anxiety for the eMotion group than the control group (adjusted mean difference −3.3, 95% CI −5.4 to −1.2). Linear regression analysis on complete data, controlling for baseline PA, revealed no between-group differences in PA at any intensity. Valid IPAQ-SF data were available for 33 trial participants at 2 months postrandomization. Linear regression analysis controlling for baseline PA revealed no between-group differences in self-reported PA.

### Sensitivity analysis

**Figure 2:**
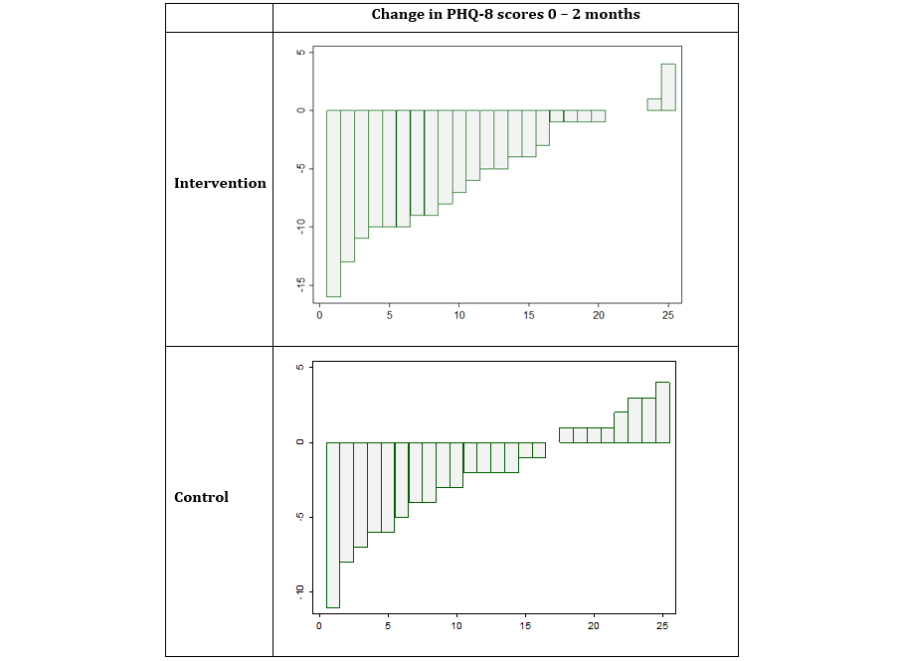
PHQ-8 change scores for individual participants.

When receipt of other psychological therapies was entered into the regression analysis as a covariate, the impact of co-treatment was not significant, and the intervention group still had a higher reduction in depressive symptoms than the control group (adjusted mean difference −3.3, 95% CI −5.9 to −0.7). Other baseline covariates (age, gender, employment, education level, and antidepressant usage) that may have influenced depression scores were also entered in the regression model together. Findings indicated that none of these variables had a significant covariate effect on depression scores and that the residual difference between groups was still significant (adjusted mean difference −3.1, 95% CI −5.7 to −0.5). Within the intervention group, linear regression analyses revealed no significant relationships between numbers of modules accessed, number of logins, or total minutes spent on the website with depression outcomes. The pattern of change scores within each group is shown in Figure 2.

**Table 4:** Between-group changes in primary and secondary outcomes.

Outcomes		eMotion		Control group		Adjusted mean difference^a^ (95% CI)
		N	Mean (SD)	N	Mean (SD)	
**Depression (PHQ-8^b^)**						
	Baseline	32	14.4 (3.4)	30	14.8 (2.9)	
	2 months postrandomization	25	8.7 (4.8)	25	12.9 (4.2)	−3.6 (−6.1 to −1.1)
**Anxiety (GAD-7^c^)**						
	Baseline	31	10.1 (5.4)	27	12.0 (4.7)	
	2 months postrandomization	25	7.1 (3.8)	25	10.9 (3.7)	−3.3 (−5.4 to −1.2)
**Min per week of objective MVPA in 10-min bouts^d^**						
	Baseline	27	29.5 (0.0 to 8.8)	25	42.1 (8.1 to 93.7)	
	2 months postrandomization	13	97.6 (49.7 to 166.3)	11	13.0 (0.0 to 131.4)	16.4 (−43.7 to 76.5)
**IPAQ-SF daily minutes of MVPA^d^**						
	Baseline	27	12.9 (0.0 to 25.7)	20	10.7 (3.6 to 17.9)	
	2 months postrandomization	19	11.4 (4.3 to 25.7)	14	15.7 (0.0 to 22.9)	0.2 (−8.7 to 9.2)

^a^Multiple regression adjusted for baseline value and confidence intervals reported.

^b^PHQ-8: Patient Health Questionnaire 8.

^c^GAD-7: General Anxiety Disorder scale.

^d^As physical activity data were skewed, medians and interquartile ranges (IQR) are presented, and analysis was repeated using bootstrapping.

## Discussion

### Summary of Findings

The present study examined the feasibility of conducting an RCT of the eMotion intervention. We successfully recruited a less-active population with elevated depression and anxiety. The trial also had acceptable attrition rates concerning the primary outcome at 2 months. Concerning the feasibility of data collection, most people provided valid accelerometer data at baseline. However, there was a lack of valid accelerometer data at 2 months postrandomization. The baseline level of MVPA in 10-minute bouts was low, with only 13% (7/52) of participants achieving at least 150 minutes of MVPA per week. Of those randomized to the intervention group, most people registered for eMotion and just over half completed the “minimum dose” (introduction, week 1, and week 2). Exploratory analyses revealed larger changes in depressive symptoms and anxiety in the eMotion group than those in the control group at 2 months postrandomization. Descriptive PA data revealed a higher weekly median of minutes of MVPA in 10-minute bouts per week in the eMotion group than in the control group at 2 months postrandomization. This difference was not significant, although this may reflect low numbers available for analysis and high variance in this measure.

### Relationship to Other Literature

The achieved recruitment rates resembled those found in other studies of Web-based interventions promoting PA for depression [29-31,45,54]. Other studies report between 26% and 46% of people approached being subsequently randomized [29-31,45,54]. Mailey et al [54] recruited students registered with mental health counseling services but did not have an inclusion criterion for the level of depressive symptoms, possibly contributing to their higher recruitment rate (46%).

Our retention rate compared well with other self-delivered trials of psychological and PA interventions for depression [30]. A recent 3-arm RCT comparing administratively supported Web-based BA, PA, and a waiting list control group had a similar retention rate to ours at 8 weeks postrandomization (82%) [30]. Conversely, a systematic review of Web-based psychological treatments for depression reported drop-out rates of 74% for unsupported, 38.4% for administratively supported, and 28% for therapist-supported treatments [38]. Some individual trials of Web-based PA interventions for depression have reported lower attrition rates than ours (8%-12%) [29,54]. However, both of these trials provided therapist support, whereas our study only provided administrative support. Our data, combined with previous research, suggested that a low level of administrative support may be adequate to retain acceptable numbers of participants at follow-up.

Most studies investigating the effect of PA on depression conceptualize PA as a prescribed structured intervention and have not previously measured baseline levels or changes in PA [6,8]. Also, change in PA has typically not been measured or reported in trials of psychological treatments for depression [55]. This is one of the few intervention studies to collect baseline PA data (using objective measures) in people with elevated depressive symptoms, building on other recent intervention studies following a similar approach [56].

With a baseline median of 35.8 (IQR 0.0-98.6) minutes per week of MVPA (in at least 10-minute bouts), we appear to have recruited a less-active sample than other similar studies. For example, a recent cross-sectional study (n=165) of adults with depression (≥10 on the PHQ-9) reported a baseline mean of 18.2 (SD 17.4) minutes of MVPA (in 10-minute bouts) per day [57]. One possible explanation for this is that the clear indication of “exercise” in the PIS attracted people with depression who were already active [57]. In eMotion, however, the intervention was not overtly presented as exercise, but rather as a behavioral intervention to promote routine, pleasurable, and necessary activities (which could include PA).

The current guidelines for PA (150 minutes of MVPA per week) are based on improving and maintaining physical health, rather than mental health [58]. The dose of PA for improving and maintaining mental health is not clear and may well be linked to the quality of the experience rather than just the physical volume. For example, a recent longitudinal cohort study (n=33,908) suggests that 12% of future cases of depression could be prevented with just 60 minutes of any intensity PA per week [59].

Only 56% of the participants in the intervention arm accessed at least the introduction, week 1, and week 2 (minimum dose), suggesting that more could be done to draw potential users into the website. eMotion actively encouraged participants to engage with the process of BA in their day-to-day lives (eg, planning and reviewing goals using their diaries). This was reflected in our process measures of receipt and enactment showing that despite the relatively low usage statistics, people randomized to eMotion were more confident to identify, select, and plan activities to improve their mood as well as to deal with setbacks (ie, to engage in the key processes of behavior change proposed by the eMotion logic model) [36]. This explanation is consistent with a recent observational study (n=8993) of a Web-based handwashing intervention (PRIMIT) [60]. In PRIMIT, the largest change in behavior occurred after the first session, with incrementally smaller changes occurring after each subsequent session [60]. Taken together, these findings suggest that usage metrics reveal little about offline engagement with intervention processes and that usage cessation could either indicate disengagement from the intervention or the development of sufficient mastery [61].

Exploratory analysis revealed a decrease in symptoms of depression and anxiety in favor of the intervention group at 2 months postrandomization, a similar reduction to that found in previous studies promoting PA for depression [23,29-31,45] and anxiety [13]. However, other studies did not find such an effect [54,62,63], possibly due to low power and the use of active control conditions [7]. Our findings tentatively support the utility of using BA as a more-general treatment for depression and anxiety and are consistent with findings from a large-scale RCT that found BA to be no less effective than cognitive behavioral therapy for treating depression [64]. However, it is important to note that due to its low power, definitive conclusions around effectiveness cannot be made from the current study.

### Strengths and Limitations

This is the first study to evaluate the feasibility of delivering BA in combination with the explicit promotion of PA, in a Web-based format. The main strength of this study was the use of rigorous methods to assess the feasibility of conducting a full-scale RCT. We used objective methods to assess PA and validated self-report measures of depression and anxiety symptoms. However, several limitations of this study need to be acknowledged.

As observed in other large-scale depression trials, our sample did not represent the wider UK population, particularly in terms of ethnicity and gender [64,65]. This is most likely an artifact of our recruitment method (ie, community recruitment) and target location (South West England). However, there are other individual factors including ability to recognize and accept mental health problems, positive impact of social networks, reluctance to discuss psychological distress and seek help among men, cultural identity, perceived social stigma against mental health, and financial factors [66]. A larger trial could attempt to recruit a more representative sample by targeting locations with more culturally diverse populations. Tailored recruitment approaches could also be used to address individual barriers to engagement (eg, using adverts targeted at males).

Although we randomized participants, due to a lack of resources for independent data collection, there was no blinding, which could have led to an inflation of the observed effects [67]. However, the potential for researcher bias was limited in this case due to an absence of any face-to-face contact when collecting outcome measures. Our groups were imbalanced at baseline with regard to co-interventions. However, these factors did not seem to strongly impact the findings. A future trial could remedy this either by including therapy as a randomization-minimization variable or adding it as an exclusion criterion.

Due to resource issues, “minimal contact” administrative support was provided by the lead author. It is possible that this may have led to bias when collecting outcomes. However, because the outcomes were collected via self-administered Web-based questionnaires and accelerometers, this is unlikely to have had an effect. We would still recommend using independent supporters in the main trial for practical reasons and for testing the feasibility of providing such support in a “real-world” NHS context, if the intervention proves effective in a full-scale RCT.

A further limitation is that the PHQ-8 was used rather than the more conventional PHQ-9. The PHQ-8 was chosen due to the lack of any directly available clinical surveillance or support for participants, as it would not have been feasible to follow-up any (Web-based) survey responses expressing suicidal ideation in response to PHQ item 9 with an immediate telephone interview. The PHQ-8 is specifically recommended for use in such circumstances [37]. Furthermore, the PHQ-8 is very similar to the PHQ-9 and has excellent convergent validity (*r*=0.997), indicating that the 2 scales are comparable [68].

### Implications for Future Research

Future studies should refine procedures (as indicated above) and further develop the eMotion intervention to optimize user engagement and experiences. Despite exploratory data showing modest reductions in depression and anxiety, only half of the people who used eMotion were mostly or very satisfied with their experience. Qualitative interviews performed on a sample of participants (n=11) have helped to identify barriers and facilitators to engaging with the intervention and with the trial (including the use of accelerometers) and suggest ways to maximize data collection and minimize attrition. This data will be reported in detail elsewhere. Refinements of the study procedures would also be needed to collect more complete and meaningful data on PA in any future trial. This could be achieved via face-to-face contact or by providing incentives.

In line with the MRC framework [69], large, well-controlled RCTs that build on the findings from this pilot trial could help to more definitively test whether such an intervention is effective in reducing depression and increasing PA in community-dwelling populations with depression in the UK and elsewhere.

### Conclusion

The eMotion intervention is novel in attempting to offer an integrated solution to the 2 critical public health priorities of depression and lack of PA. Based on the data presented, both the eMotion intervention and methods needed to conduct a trial seem to be feasible and acceptable. If successful in a large-scale trial, eMotion would have the potential to reduce depression and anxiety symptoms for people in the community, easing the burden on NHS resources. There may also be further potential to increase PA in this population.

## Abbreviations

BA: behavioral activation
BCTs: behavior change techniques
CeHReS: Centre for eHealth Research and Disease Management
CSQ-SF: Client Satisfaction Questionnaire-Short Form
GAD-7: General Anxiety Disorder scale
IPAQ-SF: International PA Questionnaire-Short Form
MVPA: moderate-to-vigorous intensity physical activity
PA: physical activity
PHQ-8: Patient Health Questionnaire 8
PIS: participant information sheet
RCT: randomized controlled trial
SMD: standardized mean difference
TIDieR: Template for Intervention Description and Replication

## Appendix

Multimedia Appendix 1 CONSORT-EHEALTH checklist (V 1.6.1).
